# The Ups and Downs of Mutation Frequencies during Aging Can Account for the Apert Syndrome Paternal Age Effect

**DOI:** 10.1371/journal.pgen.1000558

**Published:** 2009-07-10

**Authors:** Song-Ro Yoon, Jian Qin, Rivka L. Glaser, Ethylin Wang Jabs, Nancy S. Wexler, Rebecca Sokol, Norman Arnheim, Peter Calabrese

**Affiliations:** 1 Molecular and Computational Biology Program, University of Southern California, Los Angeles, California, United States of America; 2 Department of Biology, Stevenson University, Stevenson, Maryland, United States of America; 3 Departments of Genetics and Genomic Sciences, Pediatrics, and Developmental and Regenerative Biology, Mount Sinai School of Medicine, New York, New York, United States of America; 4 Departments of Neurology and Psychiatry, Columbia University Medical Center, New York, New York, United States of America; 5 Hereditary Disease Foundation, New York, New York, United States of America; 6 Department of Obstetrics and Gynecology and Department of Medicine, Keck School of Medicine, University of Southern California, Los Angeles, California, United States of America; University of Arizona, United States of America

## Abstract

Apert syndrome is almost always caused by a spontaneous mutation of paternal origin in one of two nucleotides in the fibroblast growth factor receptor 2 gene (FGFR2). The incidence of this disease increases with the age of the father (paternal age effect), and this increase is greater than what would be expected based on the greater number of germ-line divisions in older men. We use a highly sensitive PCR assay to measure the frequencies of the two causal mutations in the sperm of over 300 normal donors with a wide range of ages. The mutation frequencies increase with the age of the sperm donors, and this increase is consistent with the increase in the incidence rate. In both the sperm data and the birth data, the increase is non-monotonic. Further, after normalizing for age, the two Apert syndrome mutation frequencies are correlated within individual sperm donors. We consider a mathematical model for germ-line mutation which reproduces many of the attributes of the data. This model, with other evidence, suggests that part of the increase in both the sperm data and the birth data is due to selection for mutated premeiotic cells. It is likely that a number of other genetic diseases have similar features.

## Author Summary

Epidemiological studies show that the incidence of some genetic diseases increases with the age of the father. This “paternal age effect” is traditionally explained by the fact that, as men age, the male germ-line cells continue to divide, and each division presents an additional chance for mutation. Apert syndrome is an example of such a disease; virtually all cases are caused by spontaneous base substitution mutations of paternal origin at either one of just two sites. In this paper, we measure the frequencies of these two mutations in the sperm of unaffected men of different ages and find a frequency increase with age similar to what has been found in the data on Apert syndrome births. We also find (1) the increase in mutation frequency is not strictly monotonic, featuring a decrease followed by an increase in middle age, and (2) after normalizing for age, the two mutation frequencies are correlated within individual donors. The mutation frequency increase we observed is greater than expected based just on the number of male germ-line divisions. Along with other evidence, our data supports a novel explanation for the paternal age effect whereby Apert syndrome mutations, though harmful to the child, confer an advantage to premeiotic cells in the male germ-line that carry such a mutation. A number of other genetic diseases may exhibit similar features.

## Introduction

The paternal age effect (PAE), e.g. [1]–[5], is the phenomenon whereby the incidence of sporadic cases of certain genetic diseases increases with the age of the father. The most common explanation for this effect is that replication of premeiotic cells throughout a male's life results in the accumulation of more mutations in the germ-line of older individuals, thereby increasing the sperm mutation frequency. Given the self-renewing behavior of the dividing cell population, this explanation would suggest a linear increase in the incidence of sporadic cases with age, whereas several diseases feature an exponential increase [2],[5]. Recently, molecular tests of the PAE have been made using sperm from normal men [6]–[9]. In the case of Apert syndrome [6],[7] the results were not consistent among the different studies. Here, we possibly bridge this discrepancy. We also argue that positive selection for mutated premeiotic cells in the germ-line can explain unusual details of the relationship between the father's age and both an increase in sperm mutation frequency and the incidence of sporadic cases.

Apert syndrome (Online Mendelian Inheritance in Man #101200) is an example of a condition exhibiting a PAE. Affected individuals display prematurely fused cranial sutures and fused fingers and toes. Virtually all cases arise from a spontaneous mutation in one of two nucleotides, c.755C>G or c.758C>G (herein 755C>G and 758C>G, respectively), in the fibroblast growth factor receptor 2 gene (FGFR2) [10],[11]. In all published cases that we are aware of, the new mutations were of paternal origin [12],[13]. Since the chance of having an affected child increases exponentially with the father's age [2], it is expected that the sperm mutation frequency at these two sites should also increase exponentially with the donor's age. One study [6] found that though the frequencies of both of these mutations increase with age, their increase is not as great as expected based on the birth data [2]. These researchers tested the hypothesis that the age dependent increase in the sperm mutation frequencies in normal men is sufficient to explain the increase in birth data of affected children, and they rejected this hypothesis. However, the sensitivity of their assay may not have been sufficient to test this hypothesis. In contrast, others [7] found that by testing one of the mutations (755C>G), the frequency in sperm increases with age, and they argued that this increase is sufficient to explain their own birth data set. However, their sample size of men (especially older ones) who fathered affected children in their birth data set was rather small to statistically test the latter conclusion. Possibly explaining the disconnect between birth data and sperm data for paternal age diseases, several groups [6],[8],[9] have suggested that perhaps mutated sperm are more likely to fertilize the egg than non-mutated sperm (also see [14]).

In this study, we measure both the 755C>G and 758C>G mutation frequencies in the sperm of 314 donors, with ages ranging from 18 to 78. The number of sperm donors examined is more than double the number in any of the studies discussed in the previous paragraph. Moreover, the assays we have developed are highly sensitive, with false positive rates ∼4×10^−7^, at least 25 times lower than the earlier methods. We find that not only does the sperm mutation data show an increase with age but this increase is consistent with the birth data of affected children. We make some unusual observations in terms of a non-monotonic increase in the sperm mutation frequency with age, and the correlation between the frequencies of the two different Apert mutations in the same individuals. In addition, we apply to the observed sperm data a mathematical model for mutation accumulation in the testes, which we had previously proposed to explain the selection-based clustering of these mutations in that organ [15],[16], and consider the role that positive selection may play in explaining the Apert syndrome PAE [7].

## Results

### Birth data

**Figure 1:**
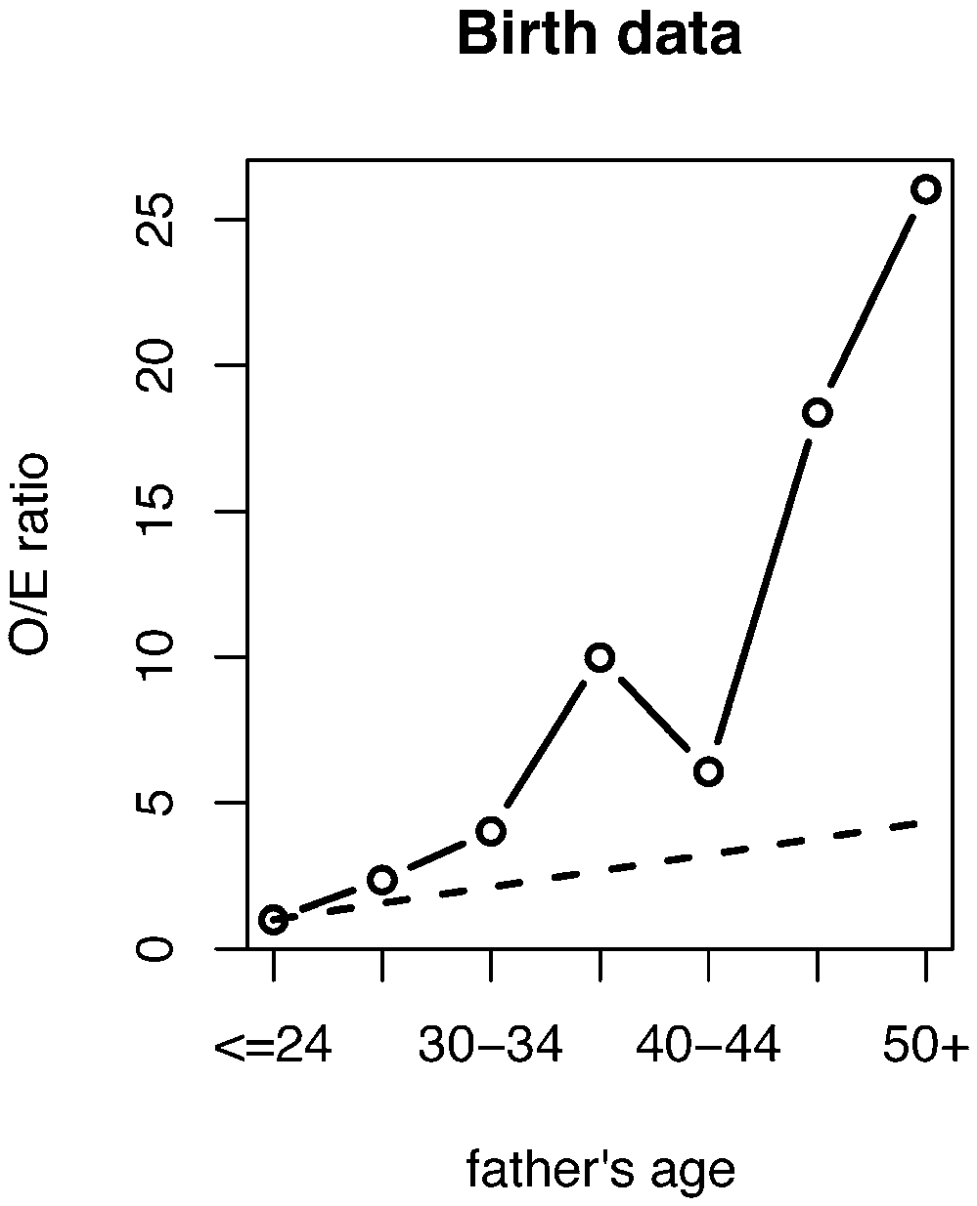
Birth data. The solid line is the observed/expected (O/E) ratio for Apert syndrome as a function of the father's age [2], normalized to be one for the youngest age category. The dashed line shows the increase expected due to the number of germ-line divisions.

The birth data [2] is a compilation of three studies published in 1960 [17], 1975 [18], and 1987 [2]. Table 1 in this paper has been copied from Table 4 in [2]. The age of the fathers has been binned into seven approximately five-year age categories: ≤24, 25–29, 30–34, 35–39, 40–44, 45–49, and ≥50 years. Figure 1 shows the observed/expected (O/E) ratio as a function of the father's age. The “observed” numerator is the number of affected births to fathers in that age category, and the “expected” denominator is proportional to the total number of births in the population (the vast majority of which do not have the disease) to fathers in that age category. The hypothesis that the sperm data is consistent with the birth data means that the number of affected births in each age category is equal to the product of the total number of births in the population for each age category and the sperm mutation frequency for that age category. Thus if this hypothesis is true, the O/E ratio is an estimate of the sperm mutation frequency.

**Table 1:** Birth data.

source		Paternal Age						
		≤24	25–29	30–34	35–39	40–44	45–49	50+
Risch 1987	Expected	7.99	8.22	5.38	2.66	1.13	0.41	0.22
	Observed	2	5	3	10	2	1	3
	O/E	0.25	0.61	0.56	3.76	1.77	2.44	13.64
Blank 1960	Expected	5.61	11.94	9.50	5.59	2.90	1.04	0.41
	Observed	1	4	9	14	5	2	2
	O/E	0.19	0.34	0.95	2.50	1.72	1.92	4.88
Cohen 1975	Expected	12.07	14.34	10.62	6.33	3.01	1.11	0.52
	Observed	3	10	12	10	3	8	2
	O/E	0.25	0.70	1.13	1.58	1.00	7.21	3.85
Total	Expected	25.67	34.50	25.50	14.57	7.04	2.56	1.15
	Observed	6	19	24	34	10	11	7
	O/E	0.26	0.55*	0.94	2.33	1.42	4.30	6.09*

Copied from reference [2] Table 4; the two entries marked with an * have been corrected from mistakes in the original.

In order to show the increase with the father's age, the O/E ratio has been scaled such that the value at the youngest age category is one. The dotted line shows the increase expected due to the number of germ-line divisions (later in this paper, we present a more detailed model). This line was calculated simply by counting the average number of germ-line divisions for fathers in each age category, using the estimate that adult self-renewing A-pale spermatogonia (SrAp) divide every 16 days [19]. These cells divide asymmetrically to create a replacement SrAp and a daughter cell whose descendents produce sperm. Thus, assuming both this division rate and the mutation rate per cell division remain constant throughout the male's life, the mutations due to replication are expected to increase linearly with age. However, the O/E ratio [2] appears to increase super-linearly, and the 26-fold increase from the youngest age category to the oldest category is greater than the 4-fold increase expected due to the number of germ-line divisions.

### Sperm data

**Figure 2:**
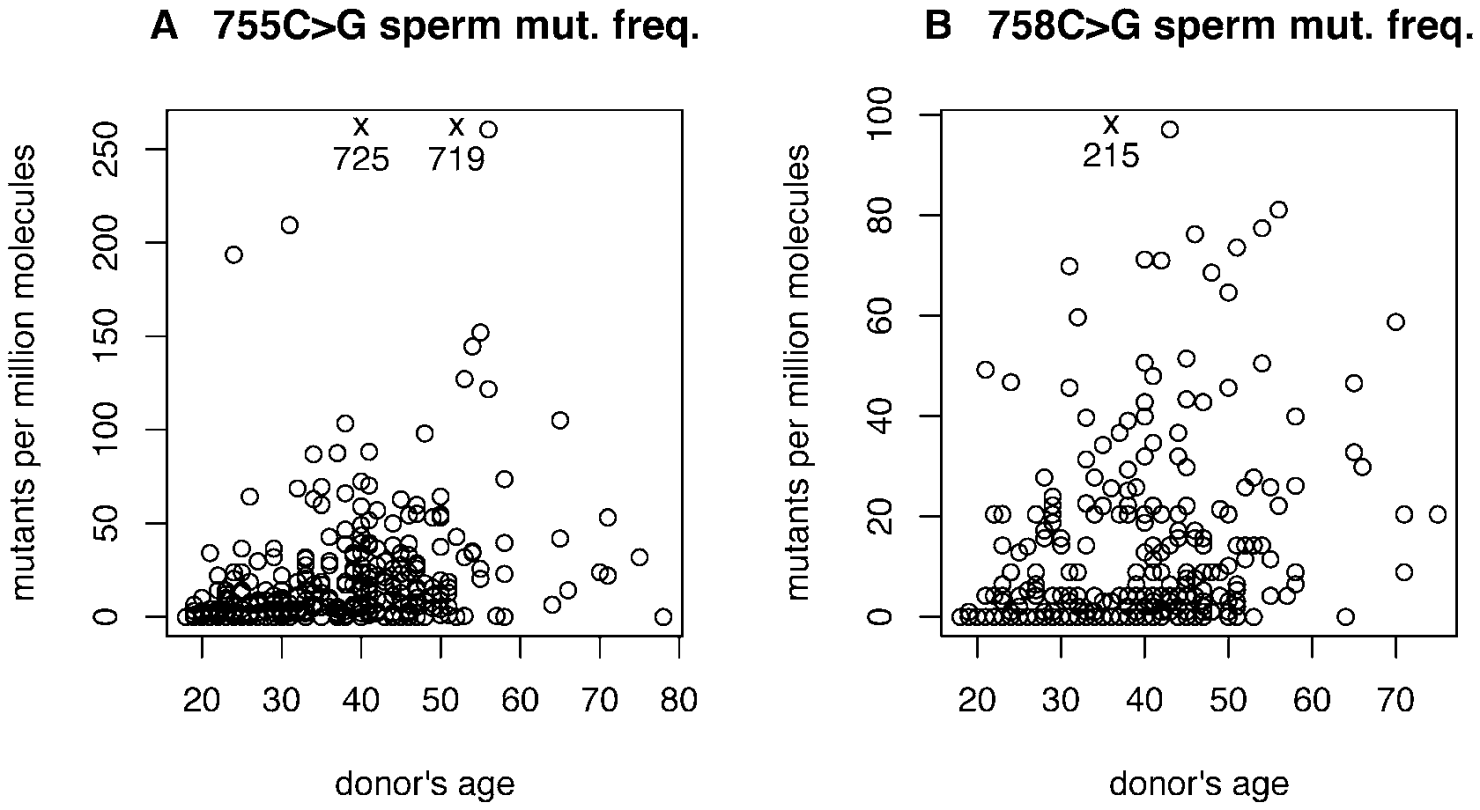
The Apert sperm mutation frequencies. (A) 755C>G and (B) 758C>G, as a function of the donor's age. Three samples with exceptionally high frequencies are marked with an X with frequency values printed below.

**Figure 3:**
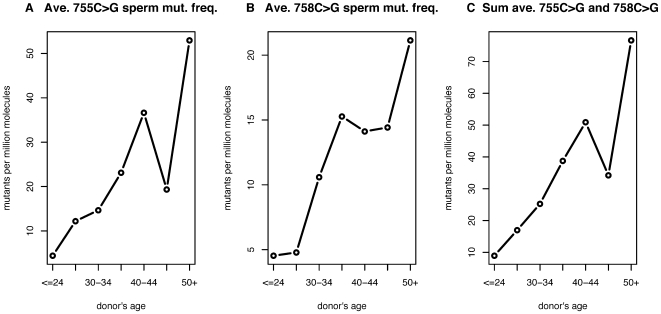
The average Apert sperm mutation frequencies. (A) 755C>G, (B) 758C>G, and (C) their sum. The age categories are the same as for the birth data.

**Figure 4:**
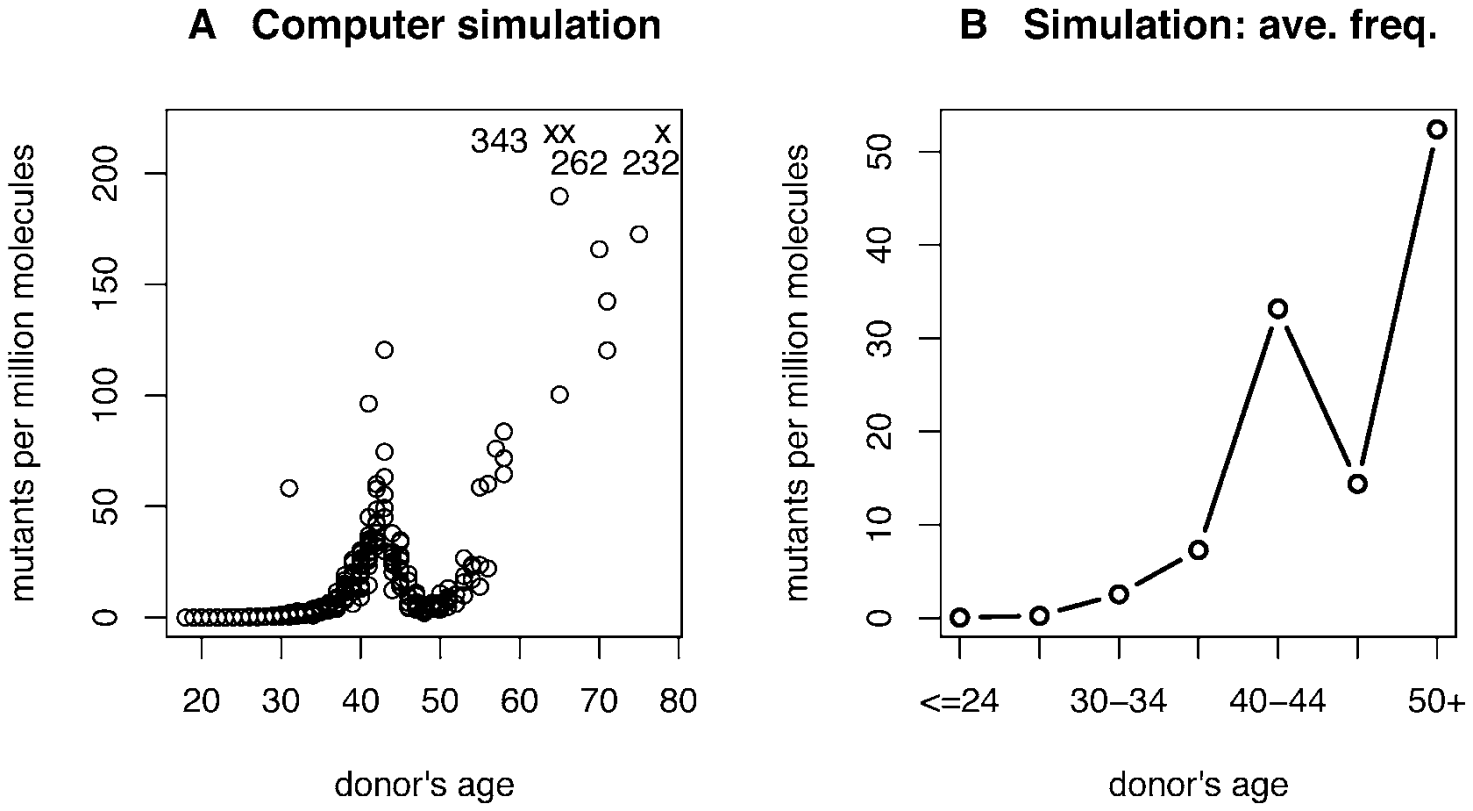
Computer simulations of the germ-line mutation model. (A) Mutation frequency as a function of age, and (B) average mutation frequency as a function of age category. Three simulations with exceptionally high frequencies are marked with an X with frequency values printed nearby.

We measured the frequency of mutations 755C>G and 758C>G in the sperm of 314 donors (for an additional 9 donors the frequency was measured for 755C>G but not for 758C>G). Figure 2A and 2B show these frequencies as a function of the donor's age (Note: the y-axis scale is different for Figure 2A and 2B, Figure 3A–3C, and Figure 4A and 4B). A spreadsheet of all the frequency estimates and 95% confidence intervals is in Table S1. The confidence intervals for each donor are relatively narrow. Quantitatively, for mutation 755C>G, for a donor with mutation frequency estimate *x* (in units of mutants per million molecules), the width of the 95% confidence interval is well approximated by 6.1+0.51*x*. To choose two illustrative examples, if the mutation frequency is 25 then the 95% confidence interval is 16 to 34, and if the mutation frequency is 100 then the 95% confidence interval is 71 to 129. The uncertainty in these measurements is considerably less than the variation between donors observed in Figure 2. Similarly, for mutation 758C>G, the width of the 95% confidence interval is well approximated by 8.7+0.80*x*. In the analyses below, we will consider both the average mutation frequency and the median mutation frequency for each age category. Due to the Law of Large Numbers, we believe the average is a more informative summary but the median is also of interest, and the conclusions do not depend on which summary is used. For both mutations, there are a few donors with mutation frequencies much higher than the other donors in their age category, and therefore, in general, the average mutation frequency for an age category is greater than the median mutation frequency for the same age category.

### Comparing sperm data to birth data

Figure 3A and 3B show the average sperm mutation frequencies for the birth data age categories. The number of sperm donors for each age category ranges from 39 to 56. Since either one of the mutations is sufficient to cause Apert syndrome, Figure 3C shows the sum of these two mutations' average frequencies.

In order to test the hypothesis that the sperm data is consistent with the birth data, we performed a likelihood ratio test, which is described in Materials and Methods and is similar to the approach described in [8]. If this hypothesis is true then the number of affected births in each age category is equal to the product of the total number of births in the population for each age category and the sum of the 755C>G and 758C>G average sperm mutation frequencies for that age category. Since there is uncertainty in the true average sperm mutation frequencies, the statistical test incorporates a bootstrap step. Based on this test, we cannot reject the hypothesis that the sperm data is consistent with the birth data (p-value 0.094). We reach the same conclusion if we use the median mutation frequency in each age category instead of the average mutation frequency (explained in more detail in Materials and Methods). We would like to emphasize that the distribution of father's ages for the total births in the population is different for the donors of contemporary semen samples compared to the earlier birth studies, but the likelihood test properly takes this change into account. However, an implicit assumption of this test, which may not be true and which we examine in the Discussion Section, is that the age-dependent increase in the sperm mutation frequency for contemporary sperm donors is similar to what would have been found for the fathers in the populations used for the birth studies.

The ratio of the average 755C>G sperm mutation frequency (across all donors) to the average 758C>G frequency is 1.99 (note the different scale in Figure 2A and 2B, and Figure 3A and 3B). This ratio is expected from clinical studies on hundreds of patients where approximately 2/3 of cases are caused by the 755C>G mutation and the remaining 1/3 by the 758C>G mutation [10],[11].

However, an examination of Figure 1 and Figure 3 shows that the general agreement between the birth data and the sperm data is not complete. For the birth data, the O/E ratio increases 26-fold from the youngest age category to the oldest. By a bootstrap procedure described in Materials and Methods, the 95% confidence interval is 7 to 100-fold. For the sperm data, the increase in the sum of the two mutations' average frequencies is only 9-fold: 13-fold for mutation 755C>G and 5-fold for 758C>G. By a separate bootstrap procedure described in Materials and Methods, the 95% confidence interval for the average sperm data increase is only 4 to 18-fold. Also for the sperm data, the increase in the sum of the two mutations' median frequencies is greater at 18-fold; the confidence interval for the medians are arbitrarily large because the median mutation frequency of the youngest age category is sometimes zero.

Further, one of the more striking visual properties of the figures is the non-monotonic increase. In Figure 3C, for the sum of the 755C>G and 758C>G mutations in sperm, the average frequency is lower for the 45–49 age category than for the 40–44 and 50+ age categories. In order to test for significance, we performed a bootstrap test described in Materials and Methods, and found that this dip had an 89% bootstrap value, which is near the significance threshold (bootstrap values greater than 95% are deemed significant). For the birth data, in Figure 1, the O/E ratio, which as described previously is expected to be a proxy for the sperm mutation frequency, is lower for the 40–44 age category than for the 35–39 and 45–49 age categories. Surprisingly, this dip is five years earlier than for the sum of the 755C>G and 758C>G average mutation frequencies in sperm. The birth data [2] is actually a collection of three separate birth studies initiated many years apart, and each one features a dip at the same age category. The bootstrap value of the birth data dip is 93%. While these bootstrap values are not significant, they suggest some difference between the sperm data and the birth data. Further, in Figure 3C, the 755C>G average mutation frequency in sperm has a dip at the same age category as the sum of the two average mutation frequencies in sperm: the bootstrap value is 96%. In Figure 3B, the 758C>G average mutation frequency shows a distinct pattern, the average frequencies for both the 40–44 and the 45–49 age categories are slightly lower than for the 35–39 and 50+ age categories. For the sperm data in this paragraph, we reach the same conclusions if we replace average mutation frequencies with median mutation frequencies.

### Correlation between mutations

Since we have measured the frequencies of the 755C>G and 758C>G mutations in the same individuals, we can compute the correlation between these frequencies. The standard correlation coefficient is statistically significant due to both mutation frequencies, on average, increasing with the donor's age. Therefore we propose a different correlation test. We fix a set of thresholds for each age category and mutation. For example, our first set of thresholds is the average mutation frequency for that age category and mutation. We count the number of donors for which both mutation frequencies are greater than their respective age-specific thresholds, and then we calculate whether this number is greater than would be expected by chance assuming that the two mutation frequencies are independent. Using the set of averages as the thresholds, by this test there is a significant correlation between the age-adjusted mutation frequencies (p-value 0.0007). We obtained similar p-values when we repeated this test using two-times-the-averages, the medians, and two-times-the-medians as the set of thresholds. A potential problem with this test is if the mutation frequency depends on age within the age categories, however we tested and did not find a significant dependence (data not shown).

### Modeling the mutation process

We have previously proposed a mathematical model for mutations in the germ-line (see [15],[16] for details). Next we review this model and consider it in relation to the data in the present study. We propose a modification to the model that might explain the non-monotonic increase, and we examine how selection for mutant premeiotic testis cells affects the PAE.

The incidence of Apert syndrome, ∼10^−5^ to 10^−6^ [20],[21], is relatively high considering that the disease is caused by one of only two possible nucleotide substitutions, and the average per nucleotide per generation mutation rate is 10^−7^–10^−9^ [22],[23]. One hypothesis is that these two nucleotides have much greater than average substitution rates (mutation hot spot); another possibility is that, while damaging to the child, these mutations provide a selective advantage to premeiotic cells in the germ-line [6]–[8],[15],[16],[24],[25]. Previously, when we divided the testes of three deceased individuals, ages 45, 54 and 62, into 192 pieces and measured the mutation frequency in each piece, we found that the mutant cells were clustered: some testis pieces had mutation frequencies 3–4 orders of magnitude greater than the rest of the testis, and 95% of the mutants were found in just 5% of the testis pieces [15],[16]. Moreover, the average mutation frequencies in these testes were relatively high, ranging from 6.7×10^−5^ to 7.3×10^−4^.

The mathematical model was originally derived to try to explain these data [15],[16]. The model has two phases. In the first phase (growth phase), cells divide symmetrically and the number of cells increases exponentially. The germ-line cells of the growth phase eventually form the self-renewing A-pale spermatogonia (SrAp). In the second phase (adult phase), beginning at puberty, the SrAp divide asymmetrically to create a replacement SrAp and a daughter cell whose descendents produce sperm. In addition, mutated SrAp occasionally divide symmetrically. The model has several parameters: the number of growth phase generations (estimated to be 30, see [15],[16]), the number of adult phase generations (depends on the individual's age, adult SrAp divide approximately every 16 days [19]), the mutation rate per cell division, and the probability the mutated SrAp divides symmetrically (called the selection parameter, if this probability is zero there is no selection). A mutation in the growth phase may establish a mutation cluster, similar to a Luria-Delbruck jackpot [26] (earlier mutations, larger clusters). If the selection parameter is zero, a mutation in the adult phase will not produce a cluster, only a single mutated SrAp lineage and the relatively small number of descendent mutant meiotic and postmeiotic cells, including sperm. However, if the selection parameter is greater than zero, the occasional symmetric division will allow the number of mutated SrAp cells to grow locally, similar to a tumor. One of the main results of our earlier papers [15],[16] is that if the selection parameter is set to zero, one cannot find a mutation rate per cell division such that the model reproduces both the observed relatively high average mutation frequency and the mutant cell clustering. However, if the selection parameter is greater than zero, one can find values for this parameter and the mutation rate per cell division such that both the observed overall frequency and the clustering are reproduced. (As further support for the selection hypothesis, when we repeated the testis dissection experiment for two younger donors, ages 19 and 23, the mutation clusters were either non-existent or had far fewer mutant cells, suggesting that the clusters in the older donors were not due to a mutation event early in development but had grown in the adult [15]. Moreover, when we assayed a different, presumably neutral nucleotide substitution at another locus in one of the older donor's testis, the mutant cells were distributed randomly without any clusters, suggesting that the disease mutations are special [16].)

Since the model simulates the spatial distribution of mutants in the testis, from this distribution we can calculate the mutation frequency for the entire testis. Based on our earlier work [15],[16], the frequency in the testis approximates the frequency of the sperm in the attached epididymis. Therefore, in this study, we compare the sperm data to a simulated data set, where for each sperm donor, we use the model to simulate two testes' frequencies, and then average these values to get the simulated sperm mutation frequency. The number of adult phase generations in each simulation depends on the age of the sperm donor; further details of the computer simulation program are discussed in Materials and Methods.

The first realization is that, without additional modification, the model cannot possibly reproduce the observed non-monotonic increase in mutation frequency with age. We address this situation by modifying the model. In the adult testis, we model the SrAp, or “self-renewing” A-pale spermatogonia, but there are also “reserve” A-dark spermatogonia (Ad) [27]. The SrAp cells regularly divide to make the precursors of sperm while the A-dark cells are for the most part quiescent [27]–[29]. However, if the number of SrAp cells is diminished, the Ad cells are thought to become active and transform into SrAp cells [28],[30]. Since Ad cells are not regularly dividing, they are thought to be less likely to have acquired any disease mutations. Therefore when mutated SrAp cells die they are possibly replaced by fresh, non-mutated Ad cells. If enough of this death and replacement occurs in middle age, it can cause a dip in mutation frequency. Figure 4 shows a simulation of the model incorporating the modifications discussed in this and the following paragraphs (details in Materials and Methods). The size of the dip in Figure 4B is similar to that observed for mutation 755C>G in Figure 3A.

The selection process produces an exponential growth of mutation clusters. This growth results in a more rapid increase of mutation frequency with age than the linear increase tied to the number of germ-line divisions for a neutral process (see Figure 1). Moreover, it allows for the dramatic increase in mutation frequency in the very next age category after the category with the decrease in frequency. As is typical with exponential processes, the amount of increase depends sensitively on the model parameters.

Two difficulties with the modified model are that (1) for older individuals the simulated sperm mutation frequencies are unrealistically high, and (2) unlike the sperm data for younger donors the simulated frequencies are all low and similar in value. In the model, after age 13 (puberty), the SrAp divide every 16 days. It seems reasonable that this cycle might lengthen as an individual ages, leading to fewer cell generations and slowing the exponential growth of mutants. However, it is difficult to estimate this cycle length in humans: to our knowledge only one experiment was ever done [19], and neither the number of volunteers for testicular injection of ^3^H and repeated biopsy, nor their ages, was given. We have modified the model in a simple way, stopping all cell generations at age 60. As we have already said, the uncertainty in the sperm mutation frequency measurements is relatively small, and therefore cannot explain the problem (2). For the simulations shown in Figure 4, all model parameters other than age are the same for all donors. We have attempted to increase the variability in simulated frequencies for younger donors by letting different donors have different parameters (randomly selected from a range of parameter values, simulations not shown) but this change did not reproduce the variability observed in the sperm data.

## Discussion

We measured the frequency of the 755C>G and 758C>G mutations in the sperm of 314 donors. Either mutation will cause Apert syndrome. On average, the frequency of both of these mutations increases with the age of the donor. We compared the actual distribution of the ages of fathers of affected children to that expected from the sperm data, namely, the normalized product of the distribution of the ages of all fathers in the population (the vast majority of whose children do not have the disease) and the observed sperm mutation frequency as a function of the donors' ages. We then used these distributions to test the hypothesis that the sperm data is consistent with the data on sporadic births of affected children in men of different ages, and we were not able to reject this hypothesis. Our results provide strong statistical support for the idea that the Apert syndrome PAE results from an increase in sperm mutation frequency. These results disagree with one paper [6], presumably due to the lack of sensitivity in that study's assay, but agree with the conclusions of another paper [7], even though these authors did not have enough data to test this hypothesis statistically. Despite this agreement, the increase from the youngest age category to the oldest age category is greater for the birth data than for the sperm data: 26-fold for the birth data and 9-fold for the average sperm mutation frequency or 18-fold for the median sperm mutation frequency. One possible explanation for this discrepancy is uncertainty in the estimates of the fold-increase for both the birth data and the sperm data. Although a selective advantage in fertilization for sperm carrying either Apert mutation over wild-type sperm is not required to explain this data, such a possibility could be another contributing factor [6],[14].

It is perhaps most clear from Figure 1, that the PAE for Apert syndrome is most likely not due only to more germ-line divisions in older males than younger males. A possible additional contributing factor is that the mutation rate per cell division may increase as men age, perhaps due to a decline in the efficiency of DNA repair mechanisms or an increase in mutagen exposure. While this mutation rate per cell division increase is possible, it would result in a uniform elevation of mutation frequencies throughout the testes. Therefore, it would not explain the clustering of cells harboring the Apert mutations in the testes of older donors that we have observed in previous publications [15],[16]. Moreover, it would not explain the apparent growth of these mutation clusters as adult men age, since when we previously studied the testes from two younger individuals, ages 19 and 23 years, the mutation clusters were either non-existent or had far fewer mutant cells than in the older donors' testes [15]. We have previously proposed a model for mutation in the male germ-line [15],[16]. This model includes positive selection, in the form of occasional symmetric divisions, on the mutant premeiotic testis cells. These occasional symmetric divisions allow the number of mutant cells to grow locally in the testis, similar to a tumor. Crow [1] independently proposed a similar selective mechanism. In this paper and our previous publications [15],[16], we estimate the mutant cells divide symmetrically approximately 1% of the time; this value is sufficient to explain both the mutation clusters observed in the older donors' testes and the growth of these clusters as adult men age. We are now arguing that this same selection scheme can also help to explain the increase in the PAE that is greater than would be expected just from the larger number of germ-line divisions in older males than younger males.

Both the sperm data and the birth data, as demonstrated by the O/E ratio, show a non-monotonic increase with age. For the birth data, researchers had previously declared this observation “difficult to explain” with any model that accumulates mutations with age during spermatogenesis [2]. These researchers suggested two possibilities: (1) heterogeneous division rates for the stem cells where those cell lines undergoing more divisions would both be more likely to harbor mutations and to die earlier, and/or (2) an introduction of fresh, undivided reserve stem cells at middle age. We have now incorporated the reserve stem cell possibility into our model for mutation in the germ-line, and simulations from the modified model reproduce the middle age decrease. In addition to this decrease, in both the sperm data and the birth data, there is a dramatic increase in frequency by the very next age category. While possibilities (1) and (2) cannot explain this dramatic increase, simulations of our model do reproduce it, due to the selection on premeiotic testis cells carrying the Apert mutations.

Not all PAE diseases feature a non-monotonic increase [2]. The type of causal mutation most likely has an effect on this property (base substitution, deletion, duplication, etc., reviewed in [31]). Interestingly, even for the Apert syndrome sperm data, the two causal base substitution mutations display different non-monotonic patterns. These two mutations are just three base pairs apart within the same gene, but subtle phenotypic differences (severity of syndactyly and the presence of cleft palate) [12],[32],[33] exist between them. In addition, functional differences between the two mutants in *in vitro* assays are known [32], [34]–[36].

The dip for the sum of the two Apert mutation frequencies in sperm is five years later than for the birth data. An implicit assumption of the generalized likelihood ratio test is that the increase in the sperm mutation frequencies for contemporary donors is similar to what would have been found for the population of fathers in the earlier birth studies. There is some evidence [37],[38] that contemporary male youths begin puberty at younger ages (resulting in more mutation events at an earlier ages), but this evidence is not conclusive [39]. Moreover, [40] report that there has been a decrease in semen quality over the last fifty years (also see [41],[42] and the contained references for more analysis). The effects of these or other possible generational changes (including environmental exposures) are not clear, but it is conceivable that they could influence the observed difference in the timing of the non-monotonic increase between the sperm data and the birth data.

In a previous publication we showed that the two Apert mutations arise independently within any one testis [15]. It was therefore unexpected that we observed a significant correlation, after normalizing for age, between the two mutation frequencies within individual sperm donors. Perhaps this correlation observation is due to some property which is heterogeneous across the population but similar for cells within a single individual. Variation in properties such as the mutation rate per cell division, the cell division rate, or the selection coefficient for cells harboring the mutations could result from genetic or environmental causes. It would be interesting to collect mutation frequencies at additional nucleotide sites from these same individuals, to examine whether some individuals have higher overall mutation frequencies than others.

Finally, achondroplasia, the most common form of dwarfism (OMIN # 100800) also shows a PAE. Almost all cases are due to a spontaneous mutation of paternal origin, at a single nucleotide site in the fibroblast growth factor receptor 3 gene (FGFR3) [43]–[46]. This disease however features a monotonic increase in both birth data [2] and sperm data [8],[9]. Also, unlike Apert syndrome, the age-dependent increase in the sperm mutation frequency for achondroplasia is not sufficient to explain the birth data ([8], for another interpretation see [8],[47]). However, additional studies are needed (including complete testis dissection analysis and the development of more sensitive assay methods) before the mechanisms behind this disease's PAE can conclusively be thought as being the same or different from Apert syndrome.

## Materials and Methods

### Sperm donors

The majority (80%) of the study semen samples were collected from partners of women undergoing evaluation for infertility at the Endocrine/Infertility Clinic of the Los Angeles County/University of Southern California Keck School of Medicine Medical Center. The study was approved by the Institutional Review Board of the University of Southern California. Other samples were recruited from the greater Baltimore area (Johns Hopkins Hospital (EWJ)) and approved by the JHH IRB. The remaining samples were recruited from men who are members of a large Venezuelan kindred, many of whom have Huntington's disease, and was approved by the Columbia Presbyterian Medical Center (CPMC) IRB (NSW).

### Molecular assays

The PAP assays [48] to measure the 755C>G and 758C>G mutation frequencies in sperm were described previously [15],[16].

### Sperm mutation frequency estimation

Initially, for each donor, 40 reactions were performed using amounts of DNA (25,000 genomes per reaction) expected to contain less than one mutant per reaction [15],[16]. If 25 or more reactions were positive, we then diluted the sample and repeated the experiment until less than 25 out of 40 positive reactions were obtained. For some donors, multiple dilutions were required. For some donors, there were multiple replicates. The maximum likelihood mutation frequency estimates and confidence intervals were calculated assuming a Poisson distribution.

### Birth data

The birth data is the “Total” entries in Table 4 of [2], which has been reproduced in this paper as Table 1.

### Likelihood ratio test

The likelihood ratio test is similar to that described in [8]. The null hypothesis is that the sperm data is consistent with the birth data. The birth data we consider is the number of affected births to fathers in each age category (the “Total: Observed” row in Table 1). Given that such a birth occurs, we calculate the probability that the father is in each of the 7 age categories under the null and alternate distributions (described below). Assuming the affected births are independent, we then calculate the likelihood of all the affected births under these two distributions and take the ratio. The null distribution is proportional to the product of the total number of births in the population for each age category (the vast majority of which do not have Apert syndrome; the “Total: Expected” row in Table 1) and the sum of the average 755C>G and 758C>G sperm mutation frequencies for that age category. The alternate distribution is the multinomial distribution inferred from the number of affected births. In order to determine the significance of this likelihood ratio, we iterate the following procedure, which simulates data under the null hypothesis while taking into account the uncertainty in the true averages of the sperm mutation frequencies: (1) simulate an affected births data set by randomly sampling from the null distribution (the simulated data set has the same number of affected births as the actual data set), (2) for each age category obtain a random average sperm mutation frequency by performing the bootstrap (with replacement) on the sperm mutation frequencies for that age category, (3) calculate a random null distribution proportional to the product of the total number of births in each age category and the random sperm mutation frequency for that category, and (4) compute the likelihood ratio for this simulated birth data set where the two distributions are the random null distribution and the multinomial distribution inferred from the simulated birth data set. Finally, we compare the likelihood ratio for the actual data to this set of simulated ratios. We also repeated this procedure using the median mutation frequencies for each age category (both when we use all the data and a random subset).

Since for the birth data we only know the age of the fathers to the resolution of the listed age categories, and since human pregnancy is approximately nine months, in order to better compare the birth data to the sperm data, we have added one year to the age of each of the sperm donors throughout this study. Further, we feel confident assuming the distribution of affected fathers' ages is uniform in each age category, as is the age of sperm donors, except for the oldest age category. Therefore, we have repeated the likelihood ratio test several times where for the oldest age category we have considered those sperm donors with ages 50–55, 50–60, 50–65, and 50+. This distinction did not affect our conclusions.

### Bootstrap

There are separate bootstrap procedures for the sperm data and the birth data. For the sperm data, for each iteration, we sample (with replacement) mutation frequencies within each age category. For each sample, we then compute the quantity of interest, namely, the average (or median) mutation frequency fold-increase from the youngest to the oldest age category, or whether or not the average (or median) frequency for the 45–49 age category is lower than for both the 40–44 and 50+ age categories. For this second quantity, the bootstrap value is the fraction of iterations for which this dip is present; we deem the result significant if the bootstrap value is greater than 95%.

For the birth data, for each iteration, we sample (with replacement) the ages of the fathers of affected children from the “Total: Observed” row in Table 1. We calculate the O/E ratio using the “Total: Expected” row in Table 1. Similar to above, for each sample, we then compute the quantities of interest on this random O/E ratio, except here we are interested in whether there is a dip such that the ratio for the 40–44 age category is lower than for the 35–39 and 45–49 age categories.

### Modeling the mutation process

The mutation model is a modification of our earlier model [15],[16]. The modified computer code can be found at the website, http://www-hto.usc.edu/people/petercal. For each individual, the model is independently simulated two times to represent the two testes; the mutation frequency in the two testes is calculated and averaged to get the simulated sperm mutation frequency. The number of adult phase generations depends on the age of the donor (*a* in years) according to the formula, 23×(*a*−13), (approximately one generation every 16 days [19] so 23 per year, starting at puberty). As discussed in the Results Section, the cell divisions are stopped at age 60. The major modification to the model is we allow SrAp cells to die and be replaced by fresh, non-mutated cells (the number of SrAp cells remains constant in the modified model). This modification introduces an additional parameter to the model: in every cell generation between the ages of 44 and 48, each SrAp (regardless of whether or not it is mutated) may die and be replaced with probability *d*. (We first tried modeling this process a different way, by letting each SrAp cell have a lifetime drawn from an exponential distribution with mean 47 years, but this did not produce the sharp dip observed in the sperm data). We searched the model-parameter space to find values such that, (1) the sperm mutation frequency for the older age categories is approximately what was observed for the 755C>G sperm mutation frequency data, (2) the magnitude of the dip was about what was observed for this data set, and (3) for donors older than age 45, the simulated spatial distribution of mutants in the testis was such that 95% of the mutants are found in approximately 5% of the testis pieces (this clustering had been observed experimentally previously [15],[16]). The parameter values in Figure 4 that satisfied these criteria are 5×10^−11^ for the mutation rate per cell division (approximately two times greater than the value estimated from testis data for the original, unmodified model without cell death and replacement [15],[16]), 0.014 for the selection parameter (similar to the value estimated from testis data for the original model [15],[16]), and 0.037 for the death probability *d* (this parameter is new to the modified model and was not in the original model). These values imply that most of the SrAp cells die and are replaced in middle age. This death and replacement does not happen all at once but is spread out among the cell generations between the ages of 44 and 48; each generation some mutated cells may be lost but the number of mutated cells may also increase due to new mutations or symmetric divisions. Therefore, in general, this process does not eliminate mutation clusters but just temporarily retards their growth. In our model when SrAp die they are always replaced, this is most likely not true, but since the number of SrAp in older individuals is no less than a third the number in younger men [49], we do not believe including this further complication would appreciably affect the model results.

## Supporting Information

Table S1 Spreadsheet of 755C>G and 758C>G sperm mutation frequencies and 95% confidence intervals for all donors and their ages.
(0.08 MB XLS)
